# DBI/ACBP is a targetable autophagy checkpoint involved in aging and cardiovascular disease

**DOI:** 10.1080/15548627.2022.2160565

**Published:** 2022-12-29

**Authors:** Léa Montégut, Adrien Joseph, Hui Chen, Mahmoud Abdellatif, Christoph Ruckenstuhl, Isabelle Martins, Frank Madeo, Guido Kroemer

**Affiliations:** a Centre de Recherche des Cordeliers, Équipe labellisée par la Ligue contre le cancer, Inserm U1138, Université Paris Cité, Sorbonne Université, Paris, France; b Metabolomics and Cell Biology Platforms, Gustave Roussy Institut, Villejuif, France; c Faculté de Médecine, Université de Paris Saclay, Kremlin Bicêtre, Paris, France; d Service de médecine intensive réanimation, Hôpital Saint-Louis, AP-HP, Paris, France; e Department of Cardiology, Medical University of Graz, Graz 8036, Austria; f BioTechMed-Graz, Graz null, Austria; g Institute of Molecular Biosciences, NAWI Graz, University of Graz, Graz, Austria; h Field of Excellence BioHealth, University of Graz, Graz, Austria; i Institut du Cancer Paris CARPEM, Department of Biology, Hôpital Européen Georges Pompidou, AP-HP, Paris, France

**Keywords:** Autophagy checkpoint, cardioprotection, heart failure, inflammation, metabolism

## Abstract

DBI/ACBP (diazepam binding inhibitor, acyl-CoA binding protein) is a phylogenetically conserved paracrine inhibitor of macroautophagy/autophagy. As such, DBI/ACBP acts as a pro-aging molecule. Indeed, we observed that the knockout of *ACB1* (the yeast equivalent of human DBI/ACBP) induces autophagy and prolongs lifespan in an autophagy-dependent fashion in chronological lifespan experiments. Intriguingly, circulating DBI/ACBP protein augments with age in humans, and this increase occurs independently from the known correlation of DBI/ACBP with body mass index (BMI). A supraphysiological DBI/ACBP level announces future cardiovascular disease (such as heart surgery, myocardial infarction and stroke) in still healthy individuals, suggesting that, beyond its correlation with chronological age, DBI/ACBP is a biomarker of biological age. Plasma DBI/ACBP concentrations correlate with triglycerides and anticorrelate with high-density lipoprotein. Of note, these associations with cardiovascular risk factors are independent from age and BMI in a multivariate regression model. In mice, we found that antibody-mediated neutralization of DBI/ACBP reduces signs of anthracycline-accelerated cardiac aging including the upregulation of the senescence marker CDKN2A/p16 (cyclin dependent kinase inhibitor 2A) and the functional decline of the heart. In conclusion, it appears that extracellular DBI/ACBP can be targeted to combat age-associated cardiovascular disease.

**Abbreviations:** BMI: body mass index; CDKN2A/p16: cyclin dependent kinase inhibitor 2A; CVD: cardiovascular disease; DBI/ACBP: diazepam binding inhibitor, acyl-CoA binding protein; ELISA: enzyme-linked immunosorbent assay; GABA: gamma-aminobutyric acid; GABR: gamma-aminobutyric acid type A receptor

## Main text

**Figure 1.**
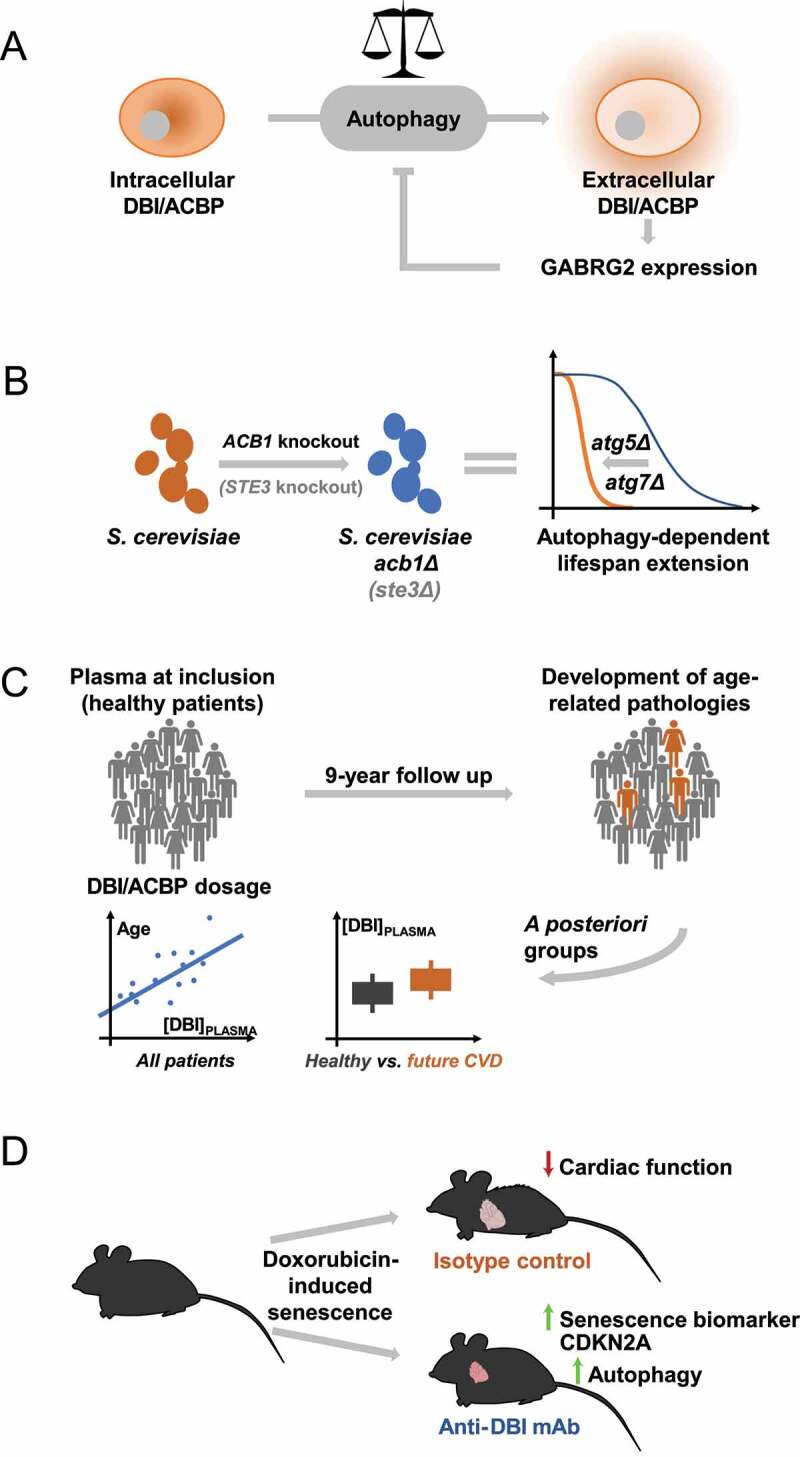
Evidence in favor of pro-aging effects of DBI/ACBP. (A) DBI/ACBP as an extracellular checkpoint of autophagy. (B) Anti-aging effects of *ACB1* knockout in yeast. (C) DBI/ACBP as a biomarker of future cardiovascular disease (CVD) in humans. (D) Anti-aging effects of DBI/ACBP neutralization in a mouse model of doxorubicin-induced cardiac aging.

DBI/ACBP is a protein with two rather distinct functions that depend on its intra- *versus* extracellular location. Within cells, DBI/ACBP binds to lipids including acyl coenzyme A esters and phosphatidylethanolamine, but DBI/ACBP can be released in the context of macroautophagy/autophagy. Extracellular DBI/ACBP then binds to benzodiazepine receptors including the GABRG2/γ2 subunit of GABR/GABA_A_R (gamma-aminobutyric acid type A receptor) to inhibit autophagy. As a result, DBI/ACBP acts as an “autophagy checkpoint” that can be targeted by genetic manipulations as well as by neutralizing monoclonal antibodies that act as “autophagy checkpoint inhibitors”. Thus, the inactivation of DBI/ACBP stimulates autophagy (Figure 1A).

It is now established that disabled autophagy constitutes one of the hallmarks of aging, based on the observations that aging is associated with a progressive decline in autophagic flux, that experimental inhibition of autophagy accelerates the aging process, and that artificial stimulation of autophagy by genetic or pharmacological interventions decelerates the pace of aging in model organisms including yeast, nematodes, flies and mice. In addition, there is indirect evidence that facets of human aging are attenuated in conditions in which autophagic flux is favored, as this is the case for leanness, physical activity, exposure to pro-autophagic micronutrients, as well as treatment with autophagy stimulatory drugs. Based on this knowledge, we recently launched a project to understand the possible role of the autophagy-inhibitor protein DBI/ACBP in aging.

Given the ease of genetic manipulation of the yeast species *Saccharomyces cerevisiae*, we first opted to knock out the yeast gene coding for the DBI/ACBP ortholog *ACB1* [1]. This genetic manipulation induces autophagic flux and increases the survival of yeast cells in chronological aging experiments, and these effects are partially mimicked by knockout of *STE3*, which codes for a pheromone receptor that also binds the Acb1 protein. Most importantly, however, we observed that the longevity-extending effect of the *ACB1* knockout was lost upon simultaneous inactivation of essential autophagy genes such as *ATG5* and *ATG7*, demonstrating a cause-effect relationship between the observed phenomena. Thus, removal of Acb1 from the system favors lifespan extension due to the induction of autophagy (Figure 1B).

In the next step, we wondered whether human aging also associates with changes in DBI/ACBP. For this, we designed an enzyme-linked immunosorbent assay (ELISA) for DBI/ACBP quantification and turned to the most accessible organ, which is peripheral blood. We took advantage of a well-annotated cohort of healthy individuals from the Loire Valley for which medical records for the subsequent decade were available. Beyond the established positive correlation between circulating DBI/ACBP and body mass index (BMI), we observed a positive association between DBI/ACBP and chronological age (measured in years) for these (still) healthy individuals. In statistical terms, this correlation between plasma DBI/ACBP concentrations and age is independent from the correlation between DBI/ACBP and BMI. Importantly, we found for those individuals that would later develop cardiovascular events (such as heart surgery, myocardial infarction and stroke) within the forthcoming 3 to 9 years that DBI/ACBP plasma levels are higher than in age- and BMI-matched controls that would remain event free (Figure 1C). This effect is observed in two different subsets of the cohort that we considered as discovery and validation datasets, suggesting that high circulating DBI/ACBP concentrations constitute a biomarker of future cardiovascular disease (CVD). Two additional observations favor this interpretation. First, in this cohort, DBI/ACBP correlates with triglycerides (which is a key risk factor of CVD) but anticorrelates with high-density lipoprotein (which protects against CVD), and these two associations are independent from age and BMI. Second, the correlation between DBI/ACBP and BMI is observable in the subset of individuals that would not develop age-associated diseases (such as CVD and cancer) but is weakened in individuals who would declare cancer and completely lost among the subset of those that would declare CVD. Altogether, these clinical results suggest that a supraphysiological elevation of DBI/ACBP constitutes a risk factor for the development of CVD and, thus, might constitute a biomarker of biological aging.

Obviously, the aforementioned clinical correlations do not allow to infer any cause-effect relationships. To prove causality, it would be necessary to design a clinical trial in which DBI/ABCP would be neutralized by antibodies or, alternatively, the interaction between DBI/ACBP and its receptor would be blocked by yet-to-be-developed small-molecule competitors. As an intermediate step, we designed a preclinical experiment in which heart aging is accelerated by systemic injection of the anthracycline doxorubicin (a chemotherapeutic agent that is well known for its dose-limiting cardiotoxicity, notably in the context of the treatment of breast cancer) and asked the question whether repeated injections of a neutralizing monoclonal antibody against mouse DBI/ACBP would attenuate the aging phenotype. We observed that DBI/ACBP neutralization induces autophagy in the heart and simultaneously reduces doxorubicin-induced signs of cardiac dysfunction such as left ventricular dilation and pulmonary congestion, as well as the upregulation of the senescence marker CDKN2A/p16 (cyclin dependent kinase inhibitor 2A). These results, in conjunction with our prior observation that blockade of DBI/ACBP attenuates myocardial ischemic injury, suggest the possible utility of neutralizing DBI/ACBP for the deceleration of cardiovascular aging (Figure 1D).

In sum, there are preclinical arguments in favor of the conjecture that DBI/ACBP may be targeted for the avoidance or deceleration of CVD. However, this hypothesis requires in-depth examination in other preclinical models of age- or stress-induced CVD, as well as a careful comparison of different methods of DBI/ACBP neutralization (such as genetic and antibody-mediated strategies) for ruling out any unwarranted off-target effects. Moreover, it appears urgent to develop antibodies able to neutralize human DBI/ACBP that eventually can be introduced into clinical trials for the treatment or prevention of age-associated diseases.
